# Lymphocytic Esophagitis Mimicking Eosinophilic Esophagitis and Esophageal Candidiasis: A Case Report

**DOI:** 10.1002/deo2.70349

**Published:** 2026-06-04

**Authors:** Hiroshi Sugawara, Makoto Eizuka, Yosuke Toya, Ryo Sugimoto, Shunichi Yanai, Yuki Yasumi, Naoki Yanagawa, Takayuki Matsumoto

**Affiliations:** ^1^ Department of Internal Medicine Division of Gastroenterology and Hepatology School of Medicine Iwate Medical University Yahaba Japan; ^2^ Department of Molecular Diagnostic Pathology School of Medicine Iwate Medical University Yahaba Japan; ^3^ Division of Gastroenterology and Hepatology Yasumi Hospital Morioka Japan

**Keywords:** endoscopic findings, eosinophilic esophagitis, esophageal candidiasis, histopathological findings, lymphocytic esophagitis

## Abstract

A 59‐year‐old man presented with a 5‐year history of progressive dysphagia. Esophagogastroduodenoscopy (EGD) revealed an esophageal stricture with adherent whitish exudates, and biopsy specimens demonstrated fungal elements. Subsequent EGD showed longitudinal furrows, concentric rings, and white exudates, mimicking eosinophilic esophagitis. Biopsy specimens revealed marked intraepithelial lymphocytic infiltration without eosinophils. Immunohistochemistry demonstrated a predominance of CD3‐positive T cells, leading to a diagnosis of lymphocytic esophagitis. Despite treatment with proton pump inhibitors, H2‐receptor antagonists, antifungal agents, and inhaled fluticasone, his symptoms persisted without sustained improvement.

## 1 Introduction

Lymphocytic esophagitis (LyE) was first described as a clinicopathological entity of chronic esophagitis by Rubio et al. in 2006 [1]. Histologically, LyE is defined by a marked increase in peripapillary intraepithelial lymphocytes, accompanied by minimal or absent intraepithelial neutrophils and eosinophils [1]. Nevertheless, consensus diagnostic criteria have not been established, and the clinical significance of LyE remains incompletely understood.

LyE often presents with non‐specific endoscopic findings, including mucosal rings, furrows, or edema, which closely resemble those observed in eosinophilic esophagitis (EoE). In addition, whitish exudates or plaques apparently mimic the appearance of esophageal candidiasis (EC) [2]. Consequently, establishing an accurate diagnosis requires careful clinicopathological correlation. We herein present a case of LyE, which was misinterpreted as EoE or EC. This case underscores the diagnostic challenges posed by LyE and highlights the critical importance of histopathological evaluation in clinical practice.

## 2 Case Report

A 59‐year‐old man visited our hospital with a 5‐year history of progressive dysphagia, which had worsened in recent months. He did not have a specific medical history, medications, allergies, or family history of gastrointestinal disease. Laboratory test revealed a white blood cell count of 6830/µL with a normal differential distribution. Hemoglobin, platelet count, and serum biochemistry were all within normal limits. Tumor markers were also unremarkable.

**FIGURE 1:**
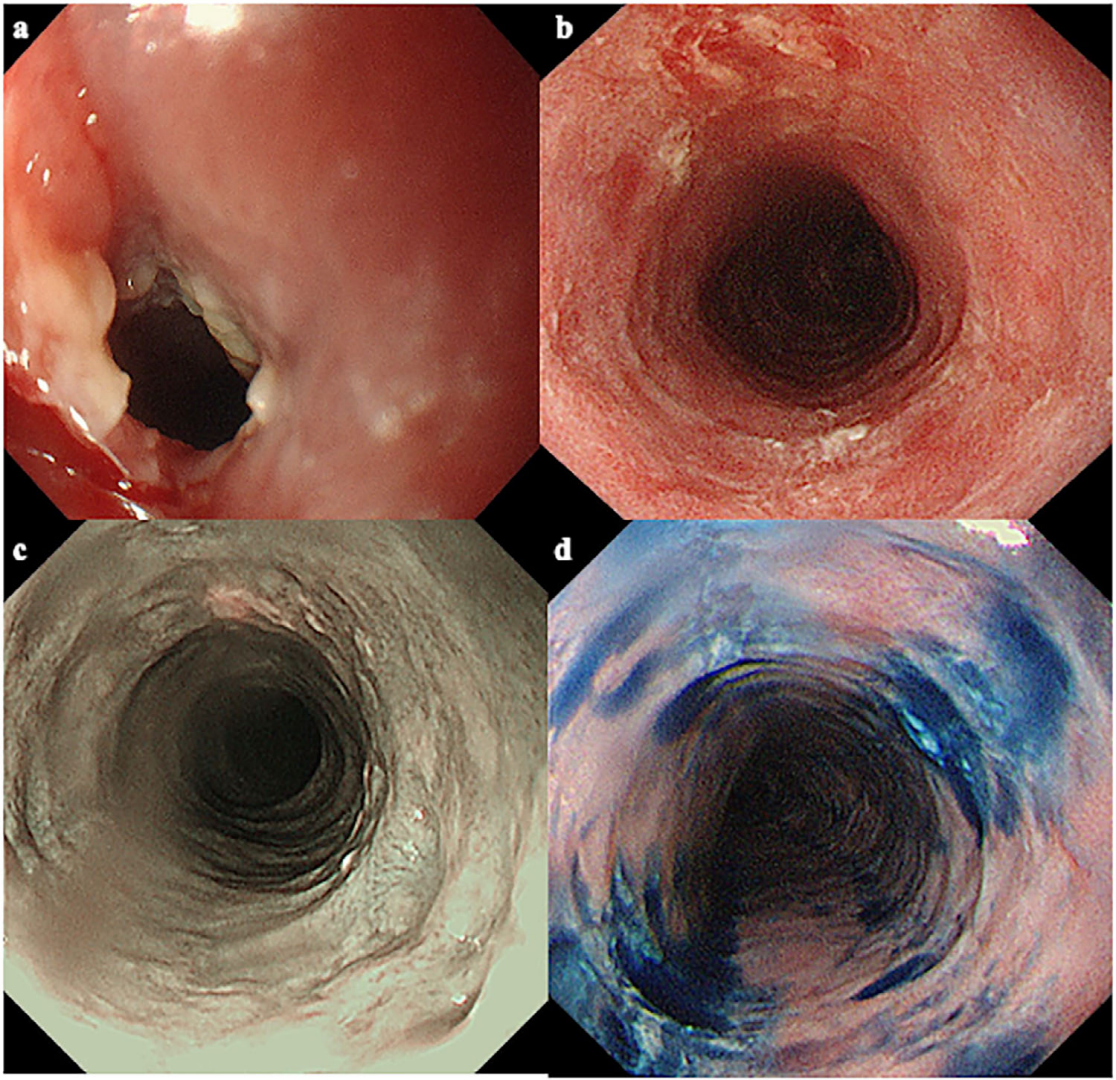
(a) White‐light imaging at the initial endoscopy revealed a stricture in the upper thoracic esophagus with adherent whitish exudates. (b) White‐light imaging beyond the stricture demonstrated concentric rings with patchy whitish exudates and scar‐like mucosal changes. (c) Narrow‐band imaging showed concentric rings with whitish exudates and the absence of cyan vessels in the esophageal mucosa. (d) Indigo carmine chromoendoscopy highlighted concentric rings in the esophagus.

**FIGURE 2:**
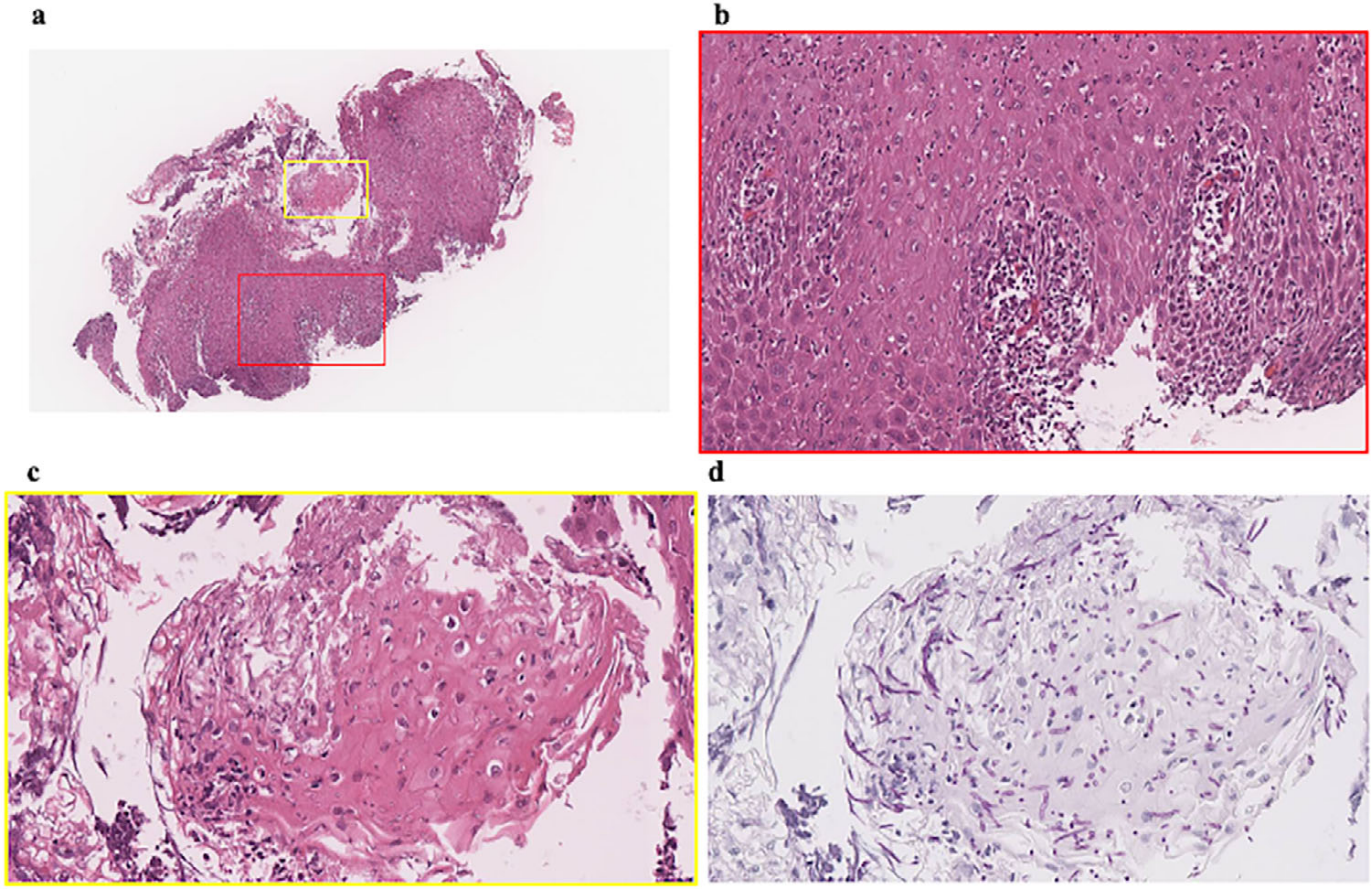
(a) Hematoxylin–eosin staining of the biopsy specimen obtained at the initial endoscopy showing intraepithelial lymphocytic infiltration (×40). (b) Hematoxylin–eosin staining showing intraepithelial lymphocytic infiltration at higher magnification (×120). (c) Periodic acid–Schiff staining demonstrating fungal hyphae (×200). d, Periodic acid–Schiff staining showing PAS‐positive fungal hyphae at higher magnification (×200).

**FIGURE 3:**
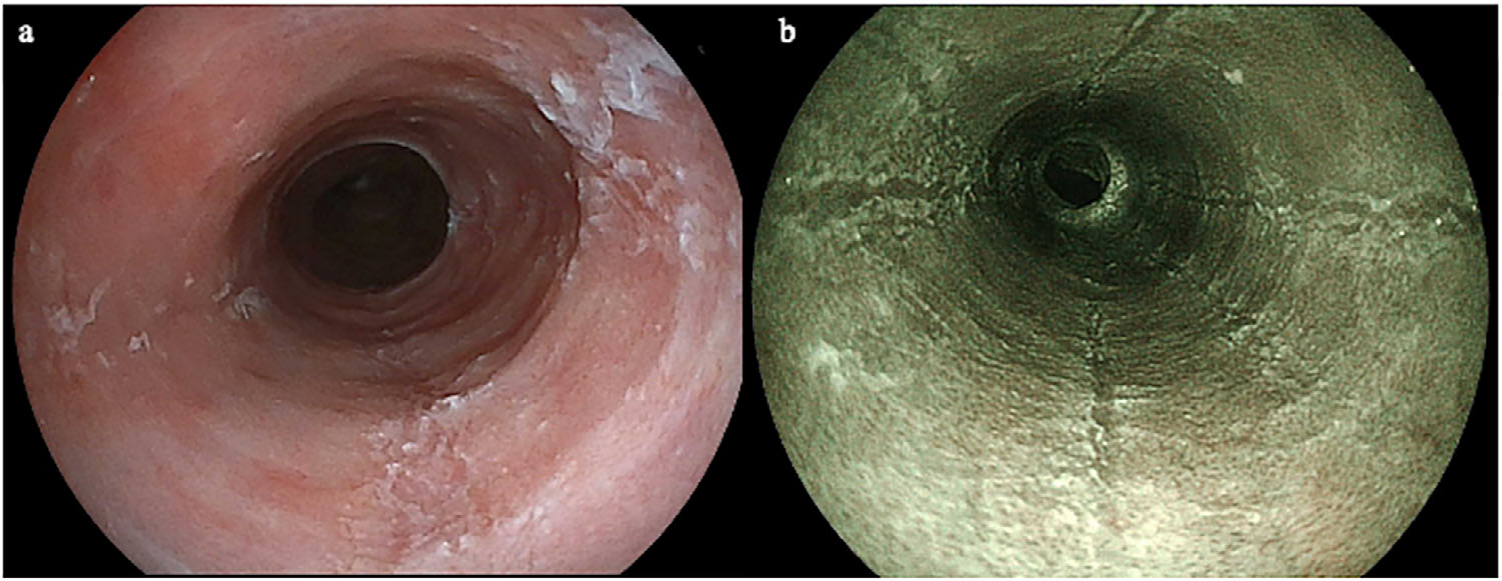
(a) White‐light imaging at two months follow‐up endoscopy showed longitudinal furrows and concentric rings with whitish exudates in the esophagus. (b) Narrow‐band imaging showed longitudinal furrows and concentric rings with whitish exudates and the absence of cyan vessels in the esophagus.

Esophagogastroduodenoscopy (EGD) using an ultrathin endoscope (GIF‐XP290N; Olympus Medical Systems, Tokyo, Japan) was performed at the initial visit. It revealed a stricture in the upper thoracic esophagus with adherent whitish exudate (Figure 1a). The endoscope could easily pass the stricture. On the anal side of the stricture, there were longitudinal furrows, concentric rings, and patchy whitish exudates with scar‐like changes (Figure 1b–d). On non‐magnifying narrow band imaging (NBI), non‐beige‐colored esophageal mucosa contained obscure dot‐shaped intrapapillary capillary loops (IPCLs) without cyan vessels. The stomach and the duodenum were found to be normal under EGD. Histopathological examination of the biopsy specimens from the upper thoracic esophagus revealed intraepithelial lymphocytic infiltration (40 lymphocytes per high‐power field [HPF]) together with fungal hyphae, which were consistent with candidiasis (Figure 2a–d). Neither band‐like superficial neutrophilic infiltration nor eosinophilic infiltration was evident. Based on these findings, the patient was diagnosed with EC, and oral amphotericin B was started. However, his symptoms did not improve. A follow‐up EGD performed two months later revealed concentric rings and longitudinal furrows, with less prominent whitish exudates when compared to the initial examination (Figure 3a,b). Biopsy specimens demonstrated intraepithelial lymphocytic infiltration but not eosinophilic infiltration. Furthermore, fungal elements had completely disappeared. Oral lansoprazole at a daily dose of 30 mg was started under a tentative diagnosis of EoE. However, his symptoms remained unchanged for the subsequent 5 years, during which repeated EGD revealed similar findings as observed at the second EGD. During the follow‐up period, there was no progression in the esophageal stricture. Various treatments, including proton pump inhibitor (PPI), H2 receptor antagonists, antifungal agents, and inhaled fluticasone, were tried, but the patient continued to experience recurrent symptom flares and remissions.

**FIGURE 4:**
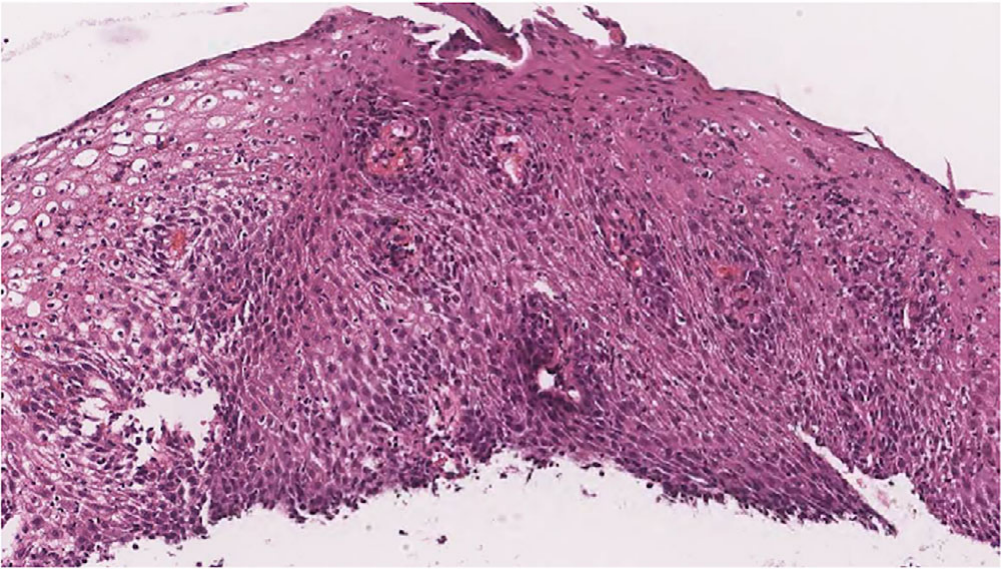
Hematoxylin–eosin staining of the biopsy specimen obtained at 5 years follow‐up endoscopy revealed intraepithelial lymphocytic infiltration without eosinophilic infiltration and fungal elements (×100).

We then re‐assessed the biopsy specimens obtained at the initial and the follow‐up EGDs. As a consequence, there were intraepithelial lymphocytic infiltrations (more than 30 lymphocytes per HPF), including involvement of the esophageal papillae (Figure 4). Immunohistochemical analysis revealed a marked increase in CD3‐positive lymphocytes. CD4‐positive lymphocytes were abundant when compared to CD8‐positive lymphocytes (Figure S1a–d). Periodic acid–Schiff staining of the follow‐up biopsy specimen showed no fungal elements (Figure S2). Based on these histopathological findings, a diagnosis of LyE was established. The patient has been under continuous PPI therapy.

## 3 Discussion

LyE represents a distinct form of chronic esophagitis characterized by intraepithelial lymphocytic infiltration [1, 2, 3, 4, 5]. Its endoscopic appearance frequently overlaps with that of EoE and EC, which may complicate the initial clinical diagnosis. In such settings, histopathological evaluation is essential for diagnostic differentiation. In contrast to EoE, standardized diagnostic criteria for LyE have not been fully established. However, previous studies proposed that marked intraepithelial lymphocytic infiltration, typically around 30–50 lymphocytes per HPF, supports the diagnosis of LyE [3]. In addition, a predominance of CD3‐positive lymphocytes has consistently been reported as a characteristic histopathological feature [2, 3].

There have been a few reports of endoscopic findings in LyE. Those reports have shown whitish exudates, longitudinal furrows, concentric rings, and esophageal strictures. Esophageal strictures have also been reported as an endoscopic manifestation of LyE, which occasionally requires endoscopic dilation in symptomatic cases [4]. However, the distribution of strictures in LyE has not been well documented, and no clear site predilection has been reported. These endoscopic findings apparently mimic those found in EoE and EC [5]. In addition to those findings under white‐light observation, Ichiya et al. reported that beige‐colored mucosa, dot‐shaped IPCLs, and absence of cyan vessels under magnifying NBI findings are characteristic of EoE and LyE [6]. Although we did not apply magnifying NBI observation to our case, cyan vessels were absent under non‐magnifying NBI.

In our case, whitish exudates even after antifungal treatment were the most significant endoscopic finding. Hussein et al. suggested that whitish exudates in LyE may reflect chronic T‐cell–mediated inflammation [7]. However, whitish exudates are also well‐recognized endoscopic features of EoE [5, 6]. Alternatively, persistent whitish exudates without the evidence of eosinophilic infiltration seem to be suggestive of LyE. In our case, however, it is also true that the stricture at the initial examination resolved after antifungal therapy without endoscopic dilation, suggesting that concomitant EC may have partly contributed to its formation.

From a clinical perspective, the management of LyE remains challenging. Current treatment strategies are largely extrapolated from those for EoE, including PPIs, topical corticosteroids, and H2‐receptor antagonists [3, 4, 5]. Nevertheless, these therapies often confer limited benefit in patients with LyE [5]. Visaggi et al. demonstrated poorer therapeutic responses and more persistent symptoms in patients with LyE compared with those with EoE despite comparable treatment regimens [5]. Another study similarly reported on the difficulty in achieving sustained clinical or histological remission in patients with LyE [3, 8]. Our case was consistent with these observations, as prolonged treatment with acid suppression, antifungal agents, and topical steroids failed to achieve sustained symptom improvement.

LyE has been reported in association with immune‐mediated conditions, including Crohn's disease, psoriasis, and celiac disease [5], raising the possibility that LyE reflects broader immune dysregulation rather than an isolated esophageal disorder. In Japan, however, LyE remains underrecognized, with only a limited number of cases reported to date [4, 9]. Increased awareness among endoscopists and pathologists, together with close clinicopathological collaboration, is essential to facilitate accurate diagnosis and to avoid unnecessary repetition of ineffective treatments.

Histologically, CD4‐positive lymphocytes were predominant in our case. It has been reported that the distribution of CD4‐ and CD8‐positive lymphocytes provides insight into the pathophysiology of LyE. Xue et al. reported that CD4‐positive T cells may predominate in cases associated with motility disorders of the esophagus, whereas CD8‐positive T cells are more common in those without motility abnormalities [10].

In conclusion, LyE should be considered in the differential diagnosis of EoE‐ or EC‐like endoscopic findings, particularly in patients who are refractory to conventional therapies. Further studies are warranted to refine diagnostic approaches and to establish disease‐specific therapeutic strategies for LyE.

## Author Contributions

**Hiroshi Sugawara**, **Makoto Eizuka**, and **Yosuke Toya** contributed to writing this manuscript. **Ryo Sugimoto**, **Shunichi Yanai**, and **Naoki Yanagawa** were involved in the patient's treatment and pathological diagnosis. **Yuki Yasumi** and **Takayuki Matsumoto** contributed to supervising the writing of the manuscript. All authors contributed to the diagnosis, the treatment, and the clinical management of the patient. All authors read and approved the final manuscript.

## Conflicts of Interest

Takayuki Matsumoto is a faculty member at the Japanese Society of Gastrointestinal Endoscopy. Yosuke Toya is the associate editor of DEN Open. The other authors declare no conflicts of interest.

## Funding

The authors have nothing to report.

## Consent

We have obtained written informed consent from the patient for the publication of this article.

## Supporting information




**FIGURE S1**: Immunohistochemistry of the biopsy specimen obtained at 5‐year follow‐up endoscopy showed positivity for CD3 (a), CD4 (b), CD8 (c), and CD20 (d), with a predominance of CD3‐positive T cells. Among these, CD4‐positive lymphocytes were more abundant than CD8‐positive lymphocytes (×100).

**FIGURE S2** Periodic acid–Schiff staining of the biopsy specimen obtained at 5‐year follow‐up endoscopy showing absence of fungal elements in the follow‐up biopsy specimen (×100).
