# Supplementary figures and images for: Lymphocytic Esophagitis Mimicking Eosinophilic Esophagitis and Esophageal Candidiasis: A Case Report
Source: DEN Open. 2026 Jun 4;7(1):e70349. doi: 10.1002/deo2.70349 (PMC13375156; doi:10.1002/deo2.70349)

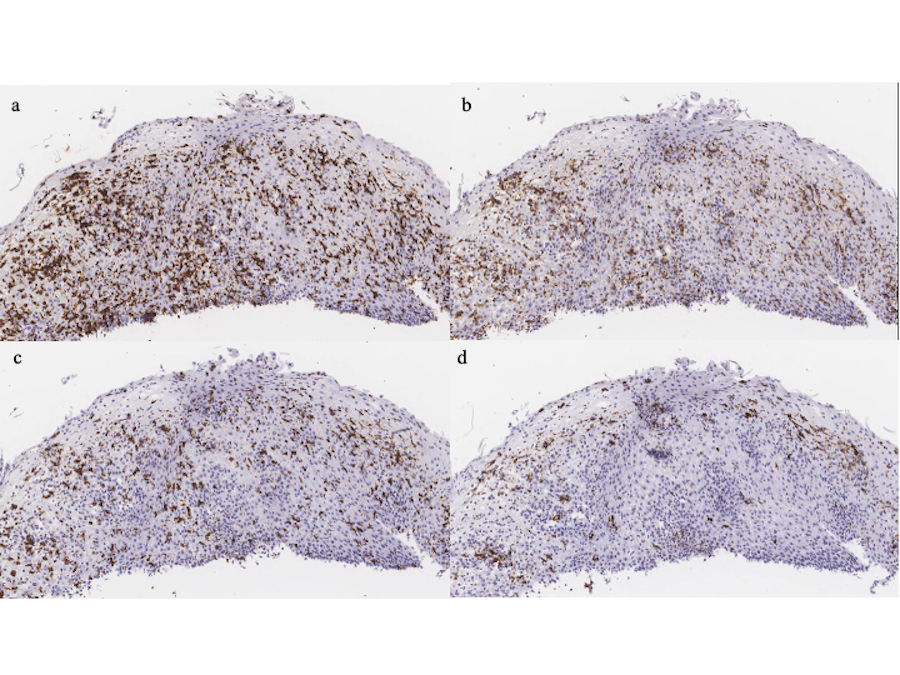

Supplement: Supplementary file 1 — FIGURE S1: Immunohistochemistry of the biopsy specimen obtained at 5‐year follow‐up endoscopy showed positivity for CD3 (a), CD4 (b), CD8 (c), and CD20 (d), with a predominance of CD3‐positive T cells. Among these, CD4‐positive lymphocytes were more abundant than CD8‐positive lymphocytes (×100). [file DEO2-7-e70349-s002.tiff]

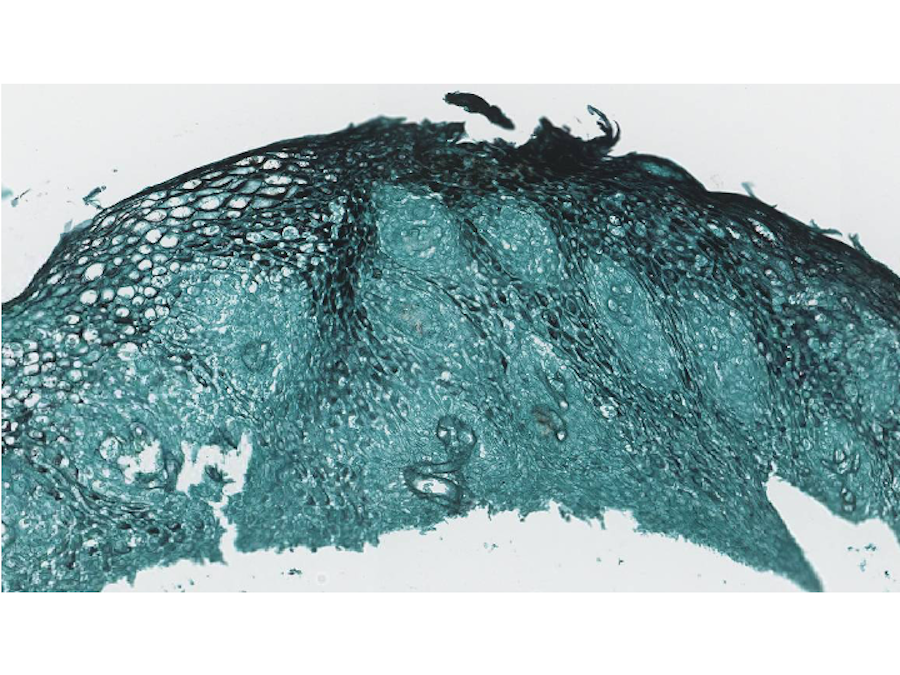

Supplement: Supplementary file 2 — FIGURE S2 Periodic acid–Schiff staining of the biopsy specimen obtained at 5‐year follow‐up endoscopy showing absence of fungal elements in the follow‐up biopsy specimen (×100). [file DEO2-7-e70349-s001.tiff]
